# P-1698. Geovirulence Factors Unique to M.bovis May Contribute to bTB Outcomes in the Mediterranean

**DOI:** 10.1093/ofid/ofaf695.1871

**Published:** 2026-01-11

**Authors:** Nicholas Foster, Liliana Salvador, Hind Azami

**Affiliations:** UGA IDIS, Athens, Georgia; University of Arizona, Tucson, Arizona; UGA IDIS, Athens, Georgia

## Abstract

**Background:**

Zoonotic tuberculosis (zTB), primarily caused by *Mycobacterium bovis*, requires a One Health approach integrating human, animal, and environmental health. To support this, we surveyed bovine tuberculosis (bTB) in Moroccan slaughterhouses and explored links to the broader Mediterranean region. Our goal was to identify virulence factors (VFs) unique to regional isolates, highlighting potential geographic variation in bTB outbreaks and their implications for human zTB transmission.

**Figure:**
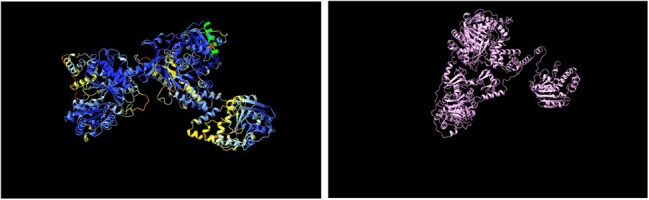
Wild Type and R584C mbtB Proteins Chimera alignment of mbtB WT and R854C (most probable conformation) mutant proteins

**Figure:**
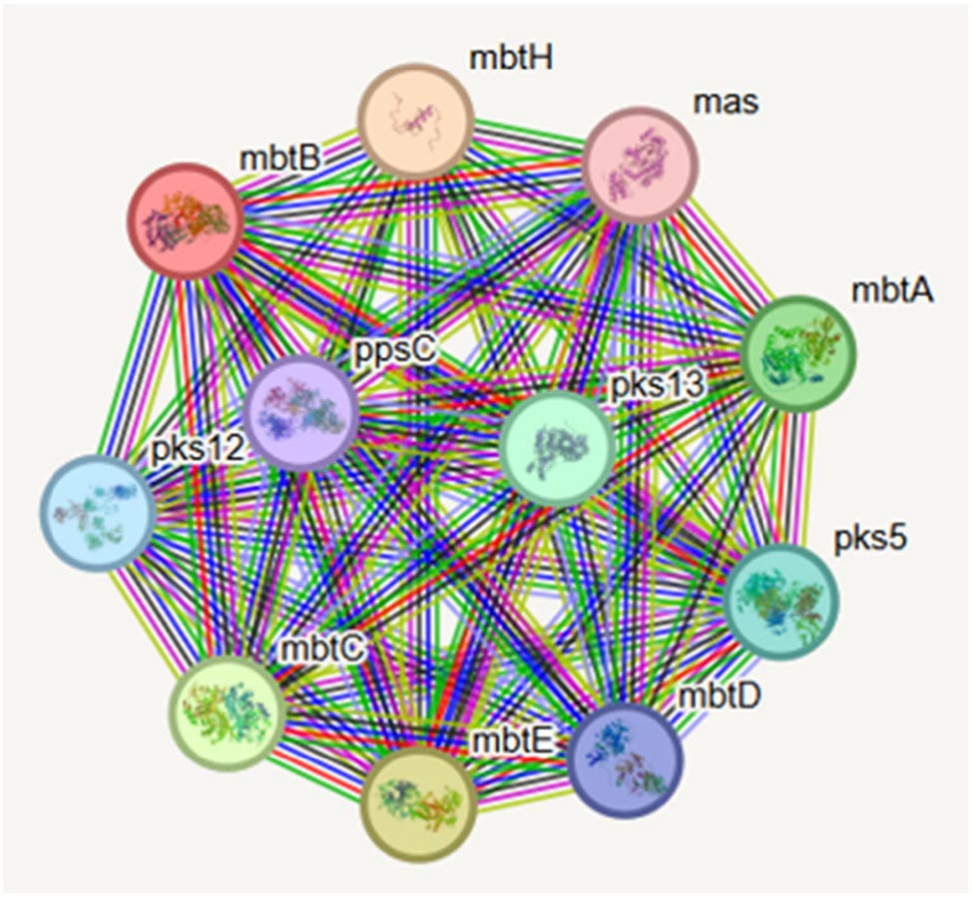
STRING Pathway Analysis of mbtB Analysis of mbtB associated proteins and pathways, implicating further downstream effector molecules and upstream cofactors.

**Methods:**

SNP calling was conducted using vSNP and R, with genome annotation and comparative analysis performed in Geneious. Chimera was used for proteomic visualization and structural modeling of predicted virulence factors.

**Figure:**
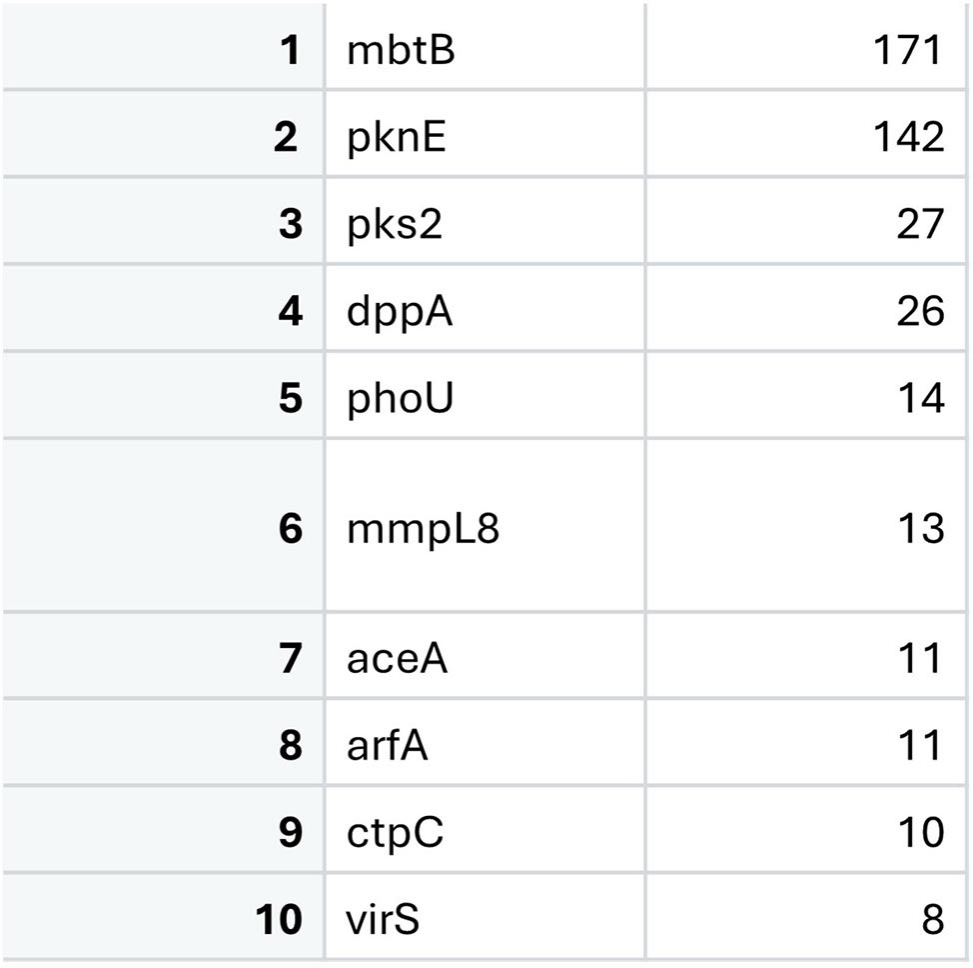
Top VF SNP Hits Top ten SNPs across the sample pool, demonstrating that mbtB R584C is present in 171 of the analyzed isolates. Other SNPs include regulatory proteins, and canonical VFs.

**Results:**

The *mbtB* locus encodes a protein essential for the iron-scavenging siderophore complex, mycobactin. Structural modeling of the R584C variant, identified in over 90% of isolates, revealed no global conformational changes (RMSD = 1.108 Å across 840 pruned atom pairs), though localized structural perturbations were noted (RMSD = 40.970 Å across 1400 pairs). R584C maps to regions with NRPS TlmIV-like adenylation, acetyl-CoA synthetase, amino acid adenylation, and AMP-binding motifs, which are involved in substrate activation and cofactor binding. This suggests the R584C substitution could alter local binding interactions or enzymatic function.

**Conclusion:**

The identification of *mbtB*, a core component of the mycobactin synthesis pathway, as a top mutational hotspot suggests selective pressure on iron acquisition systems in Mediterranean cattle populations. This may reflect an iron-limited microenvironment in bovine hosts, where efficient siderophore-mediated iron uptake is essential for *M. bovis* survival and dissemination. The high prevalence of the R584C mutation raises the possibility of a gain- or loss-of-function variant that influences bacterial fitness, with implications for zoonotic transmission and the development of strain-specific diagnostics or therapeutics. Our findings underscore the critical role of *mbtB* as a canonical virulence factor in *M. bovis* and its potential impact on bTB outcomes and zTB transmission to humans

**Disclosures:**

All Authors: No reported disclosures

